# Supplementary material for: Differential ABC transporter expression during hematopoiesis contributes to neutrophil-biased toxicity of Aurora kinase inhibitors
Source: Nat Commun. 2022 Oct 12;13:6021. doi: 10.1038/s41467-022-33672-4 (PMC9556712; doi:10.1038/s41467-022-33672-4)
Supplement: Supplementary file 1 — Supplementary Information [file 41467_2022_33672_MOESM1_ESM.pdf]

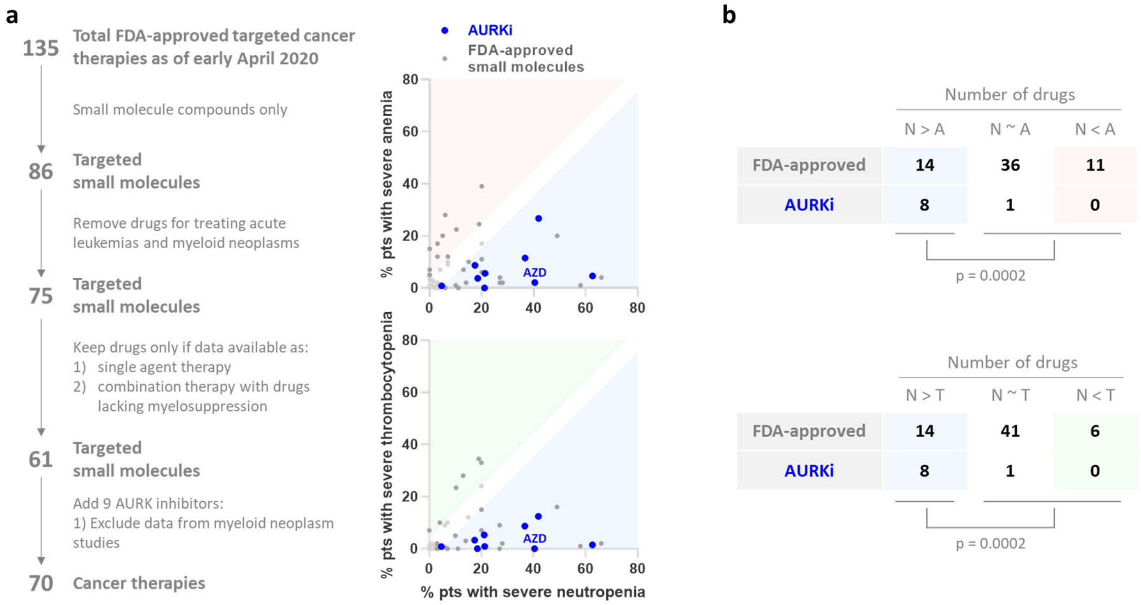

Supplementary Fig. 1 AURK inhibitors cause preferential neutropenia compared to existing FDA-approved small molecule cancer therapies. a. Rates of severe neutropenia, anemia, and thrombocytopenia were extracted from the labels of FDA-approved small molecule targeted cancer therapies for which data were available as a monotherapy or as a combination therapy with drugs known to lack myelosuppression. Drugs used in the treatment of acute leukemias or myeloid neoplasms were excluded to avoid confounding by underlying disease biology. Data from published clinical trials of AURK inhibitors (AURKi) are also included in the plots (blue dots) and AZD1152 is labeled (AZD). **b.** Tables showing number of AURK inhibitors and FDA-approved small molecule targeted cancer therapies with preferential neutropenia versus anemia (N > A) or versus thrombocytopenia (N > T), similar incidences of neutropenia versus anemia (N ~ A) or versus thrombocytopenia (N ~ T), preferential anemia (N < A), and preferential thrombocytopenia (N < T). For statistical analysis of neutropenia versus anemia bias, the “N ~ A” and “N < A” columns were combined, resulting in a 2x2 contingency table that was analyzed by Fisher’s exact test. Neutropenia versus thrombocytopenia bias was analyzed similarly. Source data are provided in the Source Data file.

	AURK inhibitor	A:B isoform selectivity	AURKA IC_{50} (nM)	AURKB IC_{50} (nM)	AURKC IC_{50} (nM)	References (PMID)
	AZD1152	**<0.0003**	1369	0.36	17	17575233
	AMG900	**0.8**	5	4	1	20935223
	AT9283	**1.0**	3	3	N/A	19143567
	MSC1992371A	**1.2**	4	4.8	6.8	19609559
	Danusertib	**6.1**	13	79	61	18089710
	PF-03814735	**6.3**	0.8	5	N/A	20354118
(E)	ENMD-2076	**25**	14	350	N/A	21177375
(T)	Tozasertib	**30**	0.6	18	4.6	14981513
(M)	MLN8237	**330**	1.2	396.5	N/A	22016509

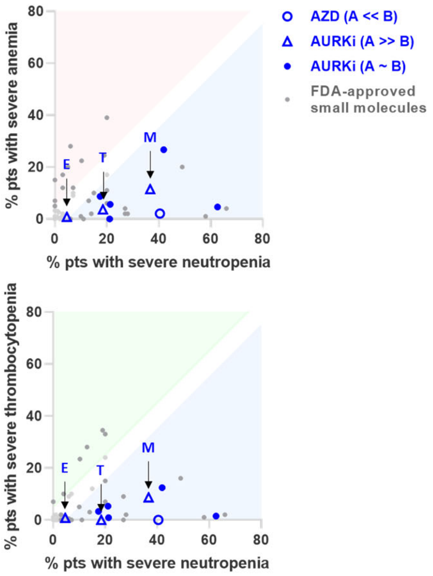

Supplementary Fig. 2 Neutropenia bias is not associated with AURK isoform selectivity of AURK inhibitors. AURK inhibitors are listed in order of increasing selectivity for AURKA over AURKB (most selective for AURKA at the bottom). The in vitro biochemical IC_{50} values of each inhibitor for AURKA, AURKB, and AURKC are shown along with their selectivity for AURKA over AURKB (AURKB IC_{50} divided by AURKA IC_{50}). The dot plots are similar to **Supplementary Fig. 1** except the most selective inhibitors of AURKA (MLN8237, tozasertib, and ENMD-2076 labeled as M, T, and E, respectively) and AURKB (AZD) are highlighted by the indicated shapes and letters. Source data are provided in the Source Data file.

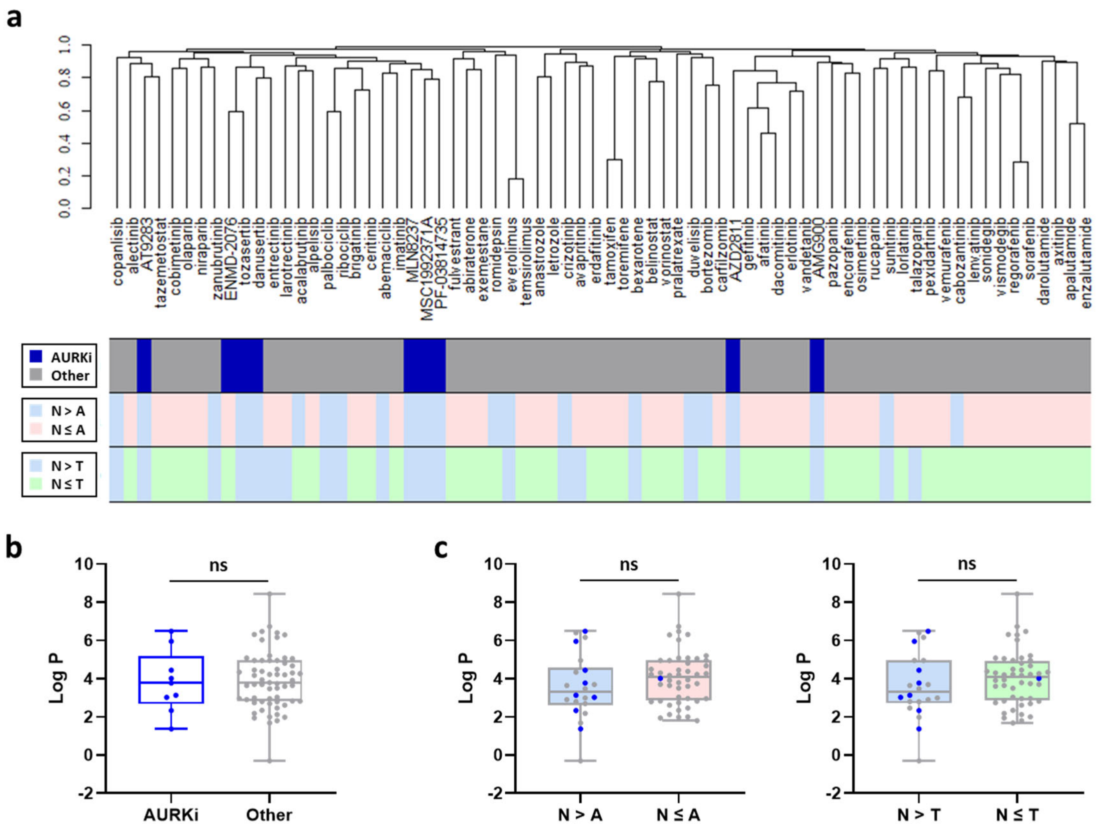

Supplementary Fig. 3 Chemical structures and lipophilicity are similarly diverse between AURK inhibitors and FDA-approved targeted cancer therapies and between drugs with and without a neutropenia bias. a. Chemical structure analysis of AURK inhibitors and FDA-approved small molecule targeted cancer therapies was performed and the dendrogram of their hierarchical clustering is shown. Y-axis indicates cluster distance. <u>Top colored bar</u>: AURK inhibitors (dark blue) do not cluster together and are thus similarly diverse to the FDA-approved drugs (gray). <u>Middle colored bar</u>: Drugs causing preferential neutropenia over anemia (N > A; light blue) do not cluster together and are thus similarly diverse to drugs that do not cause preferential neutropenia over anemia (N ≤ A; pink). <u>Bottom colored bar</u>: Drugs causing preferential neutropenia over thrombocytopenia (N > T; light blue) do not cluster together and are thus similarly diverse to drugs that do not cause preferential neutropenia over thrombocytopenia (N ≤ A; green). **b.** Calculated log P values (representing lipophilicity) are similar between AURK inhibitors and FDA-approved targeted small molecule cancer therapies. Statistical analysis based on Student's t-test. **c.** Calculated log P values are no different between drugs with and without a neutropenia-biased side effect profile. AURK inhibitors indicated by blue dots. Box plots: center line, mean; box limits, upper and lower quartiles; whiskers, minima and maxima, all data points are shown. Statistical analysis based on two-tailed Student's unpaired t-test. Source data are provided in the Source Data file.

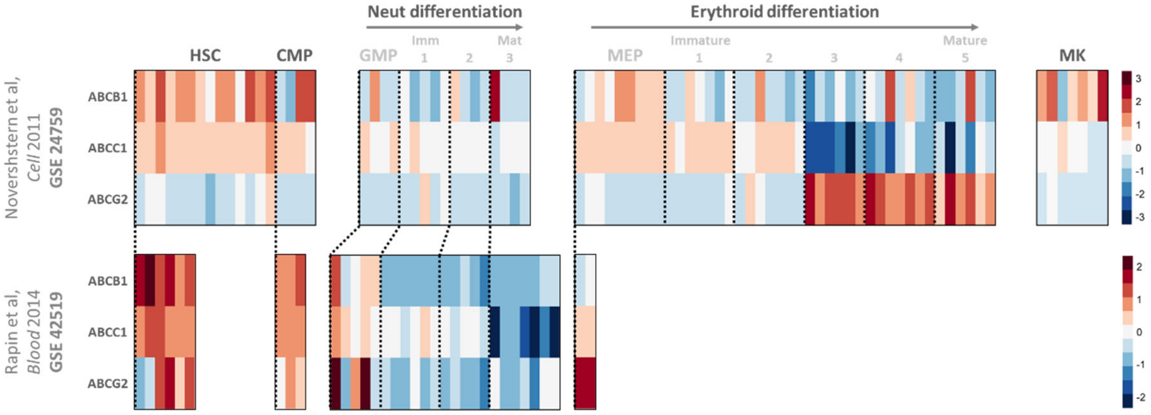

Supplementary Fig. 4 Expression patterns of ABCB1, ABCC1, and ABCG2 during hematopoietic development in 2 independent microarray datasets. Microarray data on developing human hematopoietic cells from 2 independent studies was analyzed for relative expression of ABCB1, ABCC1, and ABCG2 using the previously defined cell populations in the original studies. **HSC+MPP** (lineage$^-$ CD34$^+$CD38$^-$); **CMP** (CD34$^+$CD38$^+$CD123$^+$CD45RA$^-$); **GMP** (CD34$^+$CD38$^+$CD123$^+$CD45RA$^+$); **Neut 1** (SSC^{hi}CD11b$^-$CD16$^-$ or early + late promyelocytes); **Neut 2** (SSC^{hi}CD11b$^+$CD16$^-$ or myelocyte + metamyelocytes); **Neut 3** (SSC^{hi}CD11b$^+$CD16$^+$ or band cells + mature neutrophils); **MEP** (CD34$^+$CD38$^+$IL3Ra$^-$CD45RA$^-$); **Ery 1** (CD34$^+$CD71$^+$CD235a$^-$); **Ery 2** (CD34$^-$CD71$^+$CD235a$^-$); **Ery 3** (CD34$^-$ CD71$^+$CD235a$^+$); **Ery 4** (CD34$^-$CD71^{lo}CD235a$^+$); **Ery 5** (CD34$^-$CD71$^-$CD235a$^+$); **MK** (CD34$^-$CD41$^+$CD61$^+$CD45$^-$)

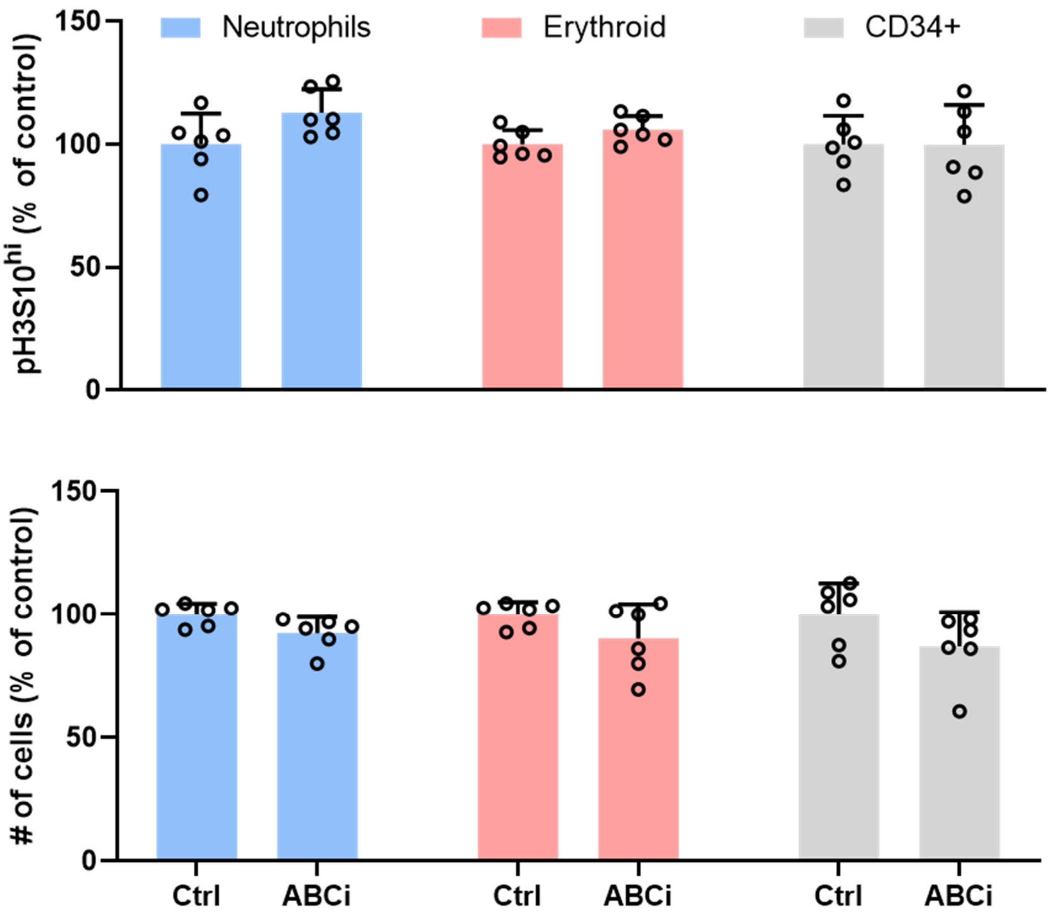

Supplementary Fig. 5 ABC transporter inhibitors do not alter pH3S10 or cell numbers. Human $CD34^+$ progenitor cells were cultured for 10 days and then treated for 48 hours with the combined inhibitors of B1 (0.5µM zosuquidar), C1 (50µM MK-571), and G2 (0.5µM Ko143) or vehicle control. Cells were harvested and cell numbers and pH3S10 were measured by flow cytometry. Mean and SD are shown; n=6, from 2 independent experiments. Source data are provided in the Source Data file.

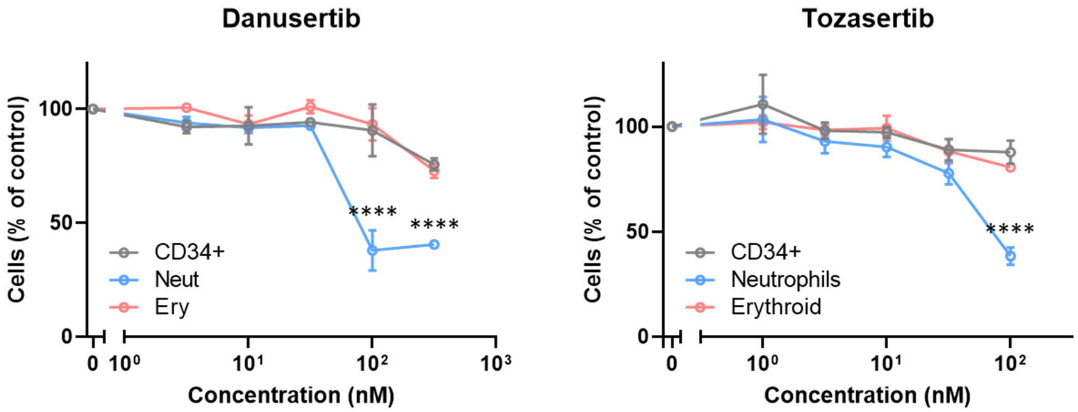

Supplementary Fig. 6 Neutrophil-biased hematotoxicity occurs with other AURK inhibitors. Human $CD34^+$ progenitor cells were cultured for 10 days and then treated for 48 hours with danusertib or tozasertib at various concentrations or vehicle control. Cells were harvested and cell numbers were measured by flow cytometry. Mean and SD are shown; n=3 for each concentration except n=1 for the vehicle control, from 1 experiment. Statistical analysis based on 2-way ANOVA of erythroid and $CD34^+$ cells compared against neutrophils at various concentrations of each respective drug. ****$p < .0001$. Source data are provided in the Source Data file.

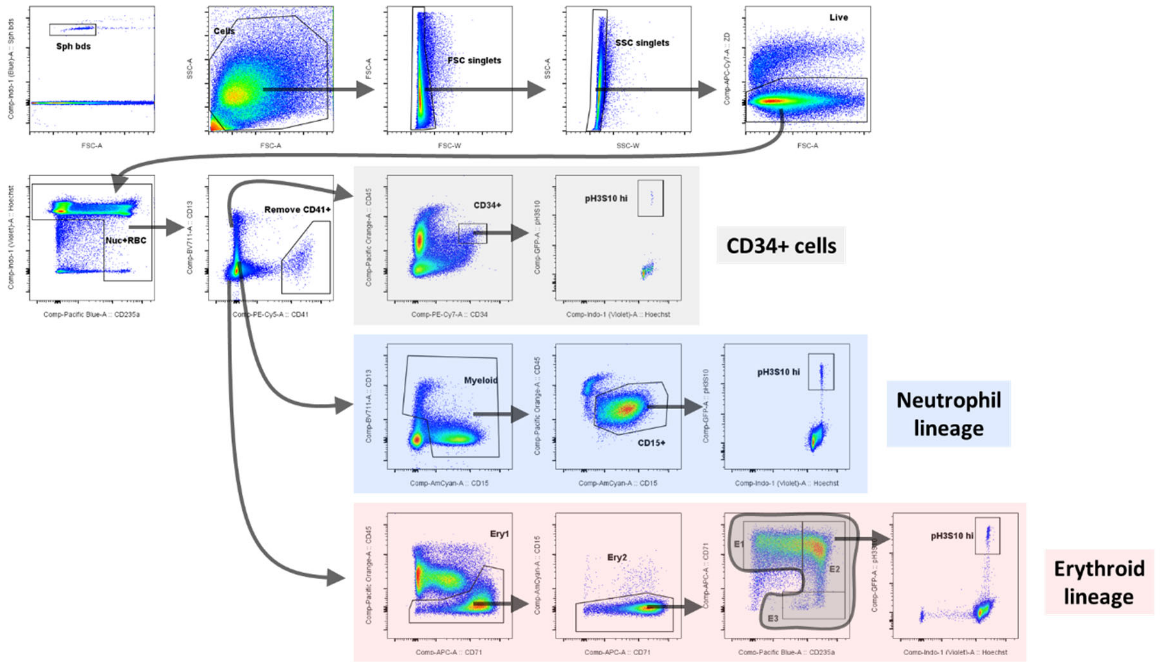

Supplementary Fig. 7 Flow cytometry gating strategy. Counting beads were gated based on FSC vs an empty channel (Indo-1 Blue). Cells were gated based on light scatter and singlet discrimination was performed based on width vs area plots for both FSC and SSC channels. Live cells were then gated on by excluding Zombie Dye positive events followed by an additional “Nuc+RBC” gate to pick out nucleated cells containing Hoechst 33342 and enucleated CD235a$^+$ RBCs. Possible CD41$^+$ megakaryocyte progenitors/debris were removed with a NOT gate and the remaining cells were split into CD34$^+$, neutrophil lineage, and erythroid lineage cells as shown. For each population, additional gates to determine cell cycle (brightness of Hoechst 33342 staining) and pH3S10 status were analyzed.
