# Supplementary material for: Differential ABC transporter expression during hematopoiesis contributes to neutrophil-biased toxicity of Aurora kinase inhibitors
Source: Nat Commun. 2022 Oct 12;13:6021. doi: 10.1038/s41467-022-33672-4 (PMC9556712; doi:10.1038/s41467-022-33672-4)
Supplement: Supplementary file 3 — Reporting Summary [file 41467_2022_33672_MOESM3_ESM.pdf]

nature portfolio

Corresponding author(s): Donald Ingber

Last updated by author(s): Aug 27, 2022

Reporting Summary

Nature Portfolio wishes to improve the reproducibility of the work that we publish. This form provides structure for consistency and transparency in reporting. For further information on Nature Portfolio policies, see our Editorial Policies and the Editorial Policy Checklist.

Statistics

For all statistical analyses, confirm that the following items are present in the figure legend, table legend, main text, or Methods section.

n/a	Confirmed	
☐	☒	The exact sample size (*n*) for each experimental group/condition, given as a discrete number and unit of measurement
☐	☒	A statement on whether measurements were taken from distinct samples or whether the same sample was measured repeatedly
☐	☒	The statistical test(s) used AND whether they are one- or two-sided<br>*Only common tests should be described solely by name; describe more complex techniques in the Methods section.*
☒	☐	A description of all covariates tested
☒	☐	A description of any assumptions or corrections, such as tests of normality and adjustment for multiple comparisons
☐	☒	A full description of the statistical parameters including central tendency (e.g. means) or other basic estimates (e.g. regression coefficient) AND variation (e.g. standard deviation) or associated estimates of uncertainty (e.g. confidence intervals)
☐	☒	For null hypothesis testing, the test statistic (e.g. *F*, *t*, *r*) with confidence intervals, effect sizes, degrees of freedom and *P* value noted<br>*Give P values as exact values whenever suitable.*
☒	☐	For Bayesian analysis, information on the choice of priors and Markov chain Monte Carlo settings
☒	☐	For hierarchical and complex designs, identification of the appropriate level for tests and full reporting of outcomes
☒	☐	Estimates of effect sizes (e.g. Cohen's *d*, Pearson's *r*), indicating how they were calculated

Our web collection on statistics for biologists contains articles on many of the points above.

Software and code

Policy information about availability of computer code

Data collection	Flow cytometry data collection: FACSDiva v8.0 (BD Biosciences)
Data analysis	Graphing and statistical analysis was performed using Graphpad Prism version 9.4.1<br>Flow cytometry data analysis: FlowJo v10 (Flowjo LLC)<br>Cheminformatics analysis was performed using Pipeline Pilot software (version 19.1.0.1964)<br>Microarray analysis was performed using R package version 1.0.12 as described in the manuscript

For manuscripts utilizing custom algorithms or software that are central to the research but not yet described in published literature, software must be made available to editors and reviewers. We strongly encourage code deposition in a community repository (e.g. GitHub). See the Nature Portfolio guidelines for submitting code & software for further information.

Data

Policy information about availability of data

All manuscripts must include a data availability statement. This statement should provide the following information, where applicable:

- Accession codes, unique identifiers, or web links for publicly available datasets
- A description of any restrictions on data availability
- For clinical datasets or third party data, please ensure that the statement adheres to our policy

All relevant raw data generated in this study are provided in the Source Data file. Previously generated microarray datasets used in this study are: GSE24759

[https://www.ncbi.nlm.nih.gov/geo/query/acc.cgi?acc=GSE24759] and GSE42519 [https://www.ncbi.nlm.nih.gov/geo/query/acc.cgi?acc=GSE42519].

Human research participants

Policy information about studies involving human research participants and Sex and Gender in Research.

Reporting on sex and gender	Cells were taken from anonymized donors without regard for sex
Population characteristics	Cells were taken from anonymized donors without regard for specific population characteristics
Recruitment	No recruitment was performed as cells were isolated from excess tissue taken as part of routine medical care
Ethics oversight	Collection of human cells was performed under Massachusetts General Hospital Institutional Review Board-approved protocol #2015P001859. Individual informed consent was not obtained as the risks to the anonymized donors were negligible. Donors were not participants in a clinical trial.

Note that full information on the approval of the study protocol must also be provided in the manuscript.

Field-specific reporting

Please select the one below that is the best fit for your research. If you are not sure, read the appropriate sections before making your selection.

☒ Life sciences ☐ Behavioural & social sciences ☐ Ecological, evolutionary & environmental sciences

For a reference copy of the document with all sections, see nature.com/documents/nr-reporting-summary-flat.pdf

Life sciences study design

All studies must disclose on these points even when the disclosure is negative.

Sample size	On the basis of prior experience, conditions routinely included 3 or 4 replicates to ensure repeatability and sufficient data points for statistical analysis; experiments were performed at least twice
Data exclusions	No data were excluded
Replication	All experiments were replicated as described in the figure legends
Randomization	Randomization is not relevant to our study as each experiment was performed on cells that were thawed from a single vial; there were no subjects/animals to randomize.
Blinding	Blinding was not performed as the experimental groups were known to the investigators in the course of conducting the experiment

Reporting for specific materials, systems and methods

We require information from authors about some types of materials, experimental systems and methods used in many studies. Here, indicate whether each material, system or method listed is relevant to your study. If you are not sure if a list item applies to your research, read the appropriate section before selecting a response.

Materials & experimental systems

n/a	Involved in the study	
☐	☒	Antibodies
☒	☐	Eukaryotic cell lines
☒	☐	Palaeontology and archaeology
☒	☐	Animals and other organisms
☒	☐	Clinical data
☒	☐	Dual use research of concern

Methods

n/a	Involved in the study	
☒	☐	ChIP-seq
☐	☒	Flow cytometry
☒	☐	MRI-based neuroimaging

Antibodies

Antibodies used	anti-CD235a-Brilliant Violet 421 (HIR2 clone; BD Biosciences; 562938; dilution: 1:100)<br>anti-CD15-Brilliant Violet 510 (W6D3 clone; BioLegend; 323028; dilution: 1:50)<br>anti-CD45-Brilliant Violet 570 (HI30 clone; BioLegend; 304034; dilution: 1:50)

anti-CD13-Brilliant Violet 711 (WM15 clone, BioLegend 301722, dilution 1:100)
anti-CD16-PE/Dazzle 594 (3G8 clone; BioLegend; 302054; dilution: 1:100)
anti-CD41-PE/Cy5 (HIP8 clone; BioLegend; 303708; dilution: 1:200)
anti-CD71-AF647 (CY1G4 clone; Biolegend; 334118; dilution: 1:200)
anti-CD34-PE-Cy7 (561 clone; BioLegend; 343616; dilution: 1:50)
anti-pHH3S10-Alexa 488 (11D8 clone; BioLegend; 650804, dilution: 1:50)

Validation

All antibodies are commonly used in the literature and have been validated by the manufacturer via quality control testing by immunofluorescent staining with flow cytometric analysis.

Flow Cytometry

Plots

Confirm that:

- [x] The axis labels state the marker and fluorochrome used (e.g. CD4-FITC).
- [x] The axis scales are clearly visible. Include numbers along axes only for bottom left plot of group (a 'group' is an analysis of identical markers).
- [x] All plots are contour plots with outliers or pseudocolor plots.
- [x] A numerical value for number of cells or percentage (with statistics) is provided.

Methodology

Sample preparation	Fresh human hematopoietic cells were isolated from leukapheresis product via magnetic beads, as described in Methods. Cultured human hematopoietic cells were harvested via pipetting and stained as described in the manuscript.
Instrument	BD LSR Fortessa
Software	Collection: FACSDiva v8.0 (BD Biosciences)<br>Analysis: FlowJo v10 (Flowjo LLC)
Cell population abundance	Cells were not sorted
Gating strategy	Counting beads were gated based on FSC vs an empty channel (Indo-1 Blue). Cells were gated based on light scatter and singlet discrimination was performed based on width vs area plots for both FSC and SSC channels. Live cells were then gated on by excluding Zombie Dye positive events followed by an additional “Nuc+RBC” gate to pick out nucleated cells containing Hoechst 33342 and enucleated CD235a+ RBCs. Possible CD41+ megakaryocyte progenitors/debris were removed with a NOT gate and the remaining cells were split into CD34+, neutrophil lineage, and erythroid lineage cells as shown. For each population, additional gates to determine cell cycle (brightness of Hoechst 33342 staining) and pH3S10 status were analyzed.

- [x] Tick this box to confirm that a figure exemplifying the gating strategy is provided in the Supplementary Information.
